# Early Anti-Drug Antibodies Predict Adalimumab Response in Juvenile Idiopathic Arthritis

**DOI:** 10.3390/ijms26031189

**Published:** 2025-01-30

**Authors:** Bo-Han Huang, Jr-Lin Hsu, Hsin-Yi Huang, Jing-Long Huang, Kuo-Wei Yeh, Li-Chen Chen, Wen-I Lee, Tsung-Chieh Yao, Liang-Shiou Ou, Syh-Jae Lin, Kuan-Wen Su, Chao-Yi Wu

**Affiliations:** 1 Division of Allergy, Asthma, and Rheumatology, Department of Pediatrics, Chang Gung Memorial Hospital, Taoyuan City 333, Taiwan; bollho@hotmail.com.tw (B.-H.H.); iuiu226633@gmail.com (H.-Y.H.); long@cgmh.org.tw (J.-L.H.); kjaecqaa@gmail.com (K.-W.Y.); leeweni@cgmh.org.tw (W.-I.L.); yaotcmail@gmail.com (T.-C.Y.); ouliangshiou@gmail.com (L.-S.O.); sjlino@adm.cgmh.org.tw (S.-J.L.); bread86@cgmh.org.tw (K.-W.S.); 2 College of Medicine, Chang Gung University, Taoyuan City 333, Taiwan; hsumike0850@gmail.com; 3 Institute of Environmental and Occupational Health Sciences, National Yang Ming Chiao Tung University, Taipei 112, Taiwan; 4 Department of Pediatrics, New Taipei Municipal TuCheng Hospital, New Taipei City 236, Taiwan; lcchen@cgmh.org.tw; 5 Department of Nursing, Chang Gung University of Science and Technology, Taoyuan City 333, Taiwan

**Keywords:** adalimumab, juvenile idiopathic arthritis, enthesitis-related arthritis, anti-drug antibodies, immunogenicity, outcome, risk factors

## Abstract

Adalimumab, a TNF-alpha inhibitor, is approved to treat juvenile idiopathic arthritis (JIA), helping control disease activity and reduce flare frequency. This study aims to investigate predictors of treatment response, including anti-drug antibodies. We reviewed 65 JIA patients (mean age 10.47 ± 3.90 years; 61.5% male) receiving adalimumab for an average of 2.64 ± 0.56 years, with demographics, laboratory parameters, therapeutic regimens, and treatment outcomes recorded. Disease status was evaluated using the Wallace criteria up to 36 months post-treatment initiation, and anti-adalimumab antibody levels were measured after 6 months of treatment. Enthesitis-related arthritis was the most common subtype (64.6%). Inactive disease status was achieved by 83.1% of patients, with 59.3% experiencing relapse. Detectable anti-adalimumab antibody at six months (*p* = 0.023) and temporomandibular joint (TMJ) involvement (*p* = 0.038) identified those less likely to achieve inactive disease. An antibody level cutoff of 7.426 ng/mL best predicted response (AUC = 0.808; *p* = 0.008), while high anti-adalimumab antibody levels after treatment (*p* = 0.032) and an injection intervals over two weeks (*p* = 0.042) were predictors of future flares. Our results highlight that the presence of anti-adalimumab antibodies six months after treatment is a risk factor for poor response to adalimumab therapy.

## 1. Introduction

Juvenile idiopathic arthritis (JIA) is a heterogeneous disease characterized by at least six weeks of arthritis in children under the age of 16. It is the most common chronic rheumatic disease in children, with an unknown etiology [1,2]. Children with chronic arthritis can develop long-term complications, including joint damage leading to physical disability and uveitis that may result in blindness, significantly affecting their quality of life [3,4]. Over the past two decades, the introduction of biological disease-modifying anti-rheumatic drugs (DMARDs) has significantly improved the prognosis for patients with JIA [5]. These treatments have enabled more children to reach adulthood without severe joint damage or complications from persistent uveitis, especially for those who do not respond well to or cannot tolerate conventional DMARDs, such as methotrexate (MTX) [5,6,7].

Adalimumab is a fully human monoclonal IgG1 antibody against tumor necrosis factor that has been approved for the treatment of JIA since 2008 and is now widely recommended by experts [8,9,10]. It has been found to be well-tolerated and generally safe, showing good efficacy in treating JIA while reducing joint pain and benefiting patients with uveitis [6]. Although many patients experience significant clinical improvement, full clinical remission is not achieved by all [11,12,13]. Studies show remission rates can reach up to 74.7% at 24 weeks, particularly in those with a polyarticular course [13]. However, patients with enthesitis-related arthritis (ERA) often continued to experience active disease despite treatment [14,15]. While various studies have explored clinical factors linked to adalimumab response, consistent findings are still lacking.

Besides clinical factors, immunogenicity with the production of anti-drug antibodies has been linked to reduced serum drug concentrations and decreased efficacy of biological DMARDs [16,17,18]. Compared to adults, pediatric patients exhibit an earlier immunogenic response to adalimumab [19]. Studies have found a significant proportion of JIA patients develop anti-adalimumab antibodies following treatment [16,17,18,20,21]. Given that these antibodies can be detected as early as 2–3 months following treatment and reach its peak presence around 6 months [22], this raises the question of whether anti-adalimumab antibodies might serve as early predictors of therapeutic response.

In the present study, we aim to explore the factors associated with the response to adalimumab treatment, with special interest in the predictive role of anti-adalimumab antibodies detected in the patient serum 6 months following treatment.

## 2. Results

### 2.1. Patients’ Characteristics

A total of 65 patients with JIA treated with adalimumab underwent regular outpatient follow-ups for symptoms, laboratory tests, and medications. Among them, there were 40 male subjects and 25 female subjects. The mean age at time of JIA diagnosis was 10.47 ± 3.90 years, and the duration of disease follow up was 7.97 ± 5.53 years. Overall, the duration between diagnosis and initiation of adalimumab was 3.28 ± 4.97 years, and the mean duration of adalimumab use was 2.64 ± 0.56 years. Among the patients, the most common classification of JIA was ERA (*n* = 42, 64.6%), followed by seronegative polyarthritis (*n* = 16, 24.6%). Prior to the application of adalimumab, four patients received etanercept, a recombinant soluble TNF receptor fusion protein acting as a TNF inhibitor. Baseline characteristics as well as detailed medication history before the initiation of adalimumab are shown in Table 1.

### 2.2. Comparison Between the Response and Non-Response Groups

**Figure 1:**
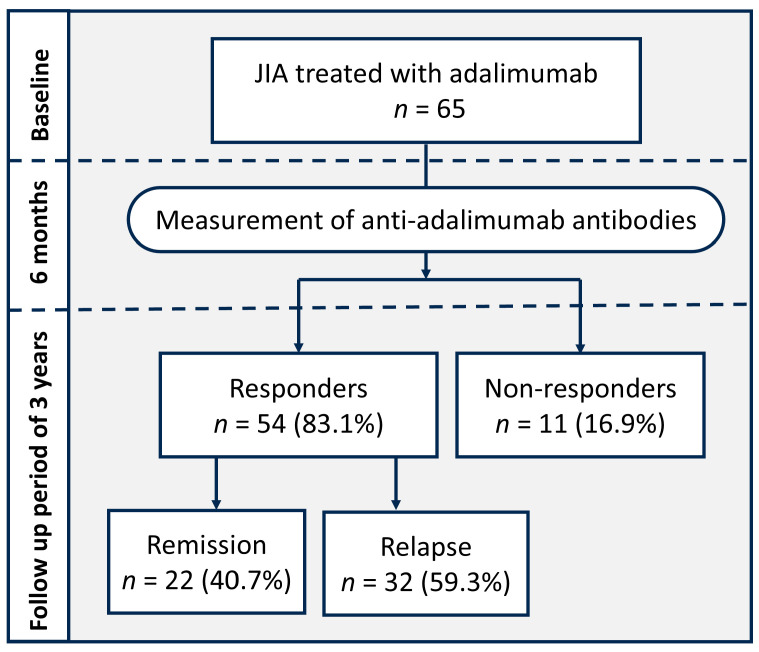
Flow chart with overview of patients’ response to adalimumab treatment.

Disease activity was assessed every 6 months up to 36 months after adalimumab initiation. The levels of anti-adalimumab antibodies 6 months after treatment were obtained from 50 (76.9%) of the cases. During the follow-up period of 36 months, fifty-four (83.1%) patients achieved at least one instance of inactive disease status, and were categorized into the “response group”. Among them, twenty-two (40.7%) patients remained in inactive disease status until the end of follow-up. Thirty-two (59.3%) of them who did not fulfill the definition of “remission” following an initial inactive disease status were categorized as “relapse”. The grouping of patients with different therapeutic responses to adalimumab was illustrated in Figure 1.

Comparisons between the “response group” and the “non-response group” are presented in Table 2. In the present cohort, more than 95% of the patients received MTX as concomitant medications in either group during the follow up period. While no differences were noted in sex, age at diagnosis, duration from diagnosis to adalimumab initiation, or duration of adalimumab use, patients with temporomandibular (TMJ) joint involvement were less likely to achieve inactive disease status (*p* = 0.038). Considering that ERA has been reported to have a low remission rate, we also looked into the response of ERA and non-ERA patients to adalimumab treatment and found that the response rate to adalimumab was not inferior in patients with ERA as compared to other JIA subtypes (*p* = 1.000). Additionally, twenty-five out of 50 (50%) of the cases had detectable anti-adalimumab antibodies. The presence of anti-adalimumab antibodies was significantly associated with a reduced therapeutic response, as a higher proportion of patients with detectable anti-adalimumab antibodies was observed in the “non-response group” compared to the “response group” (73.7% vs. 31.5%, *p* = 0.023).

### 2.3. Role of Anti-Drug Antibodies in Adalimumab Treatment Response Prediction

**Figure 2:**
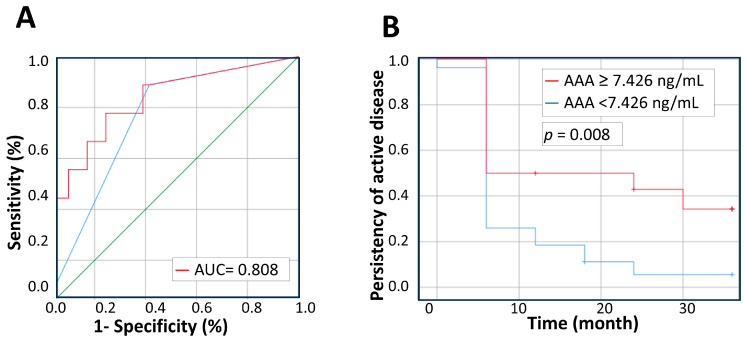
Predicted value of anti-adalimumab antibodies in treatment responses after 6 months of adalimumab treatment among JIA patients. (**A**) ROC curve of anti-adalimumab antibodies for JIA patients with persistent active disease following adalimumab treatment. The red line depicts a cutoff value of 7.426 ng/mL of anti-adalimumab antibody and the blue line depicts a cutoff value of 0. (**B**) Kaplan–Meier survival curve for persistency of active disease among JIA patients with a cutoff value of 7.426 ng/mL of anti-adalimumab antibody. The red line consisted of patients whose serum concentration of anti-adalimumab antibodies exceeded this threshold, while the blue line consisted of those below it.

Considering the correlation between anti-adalimumab antibodies and the therapeutic response, we further evaluated whether there is a better cutoff value of the anti-adalimumab antibody titer other than 0 in differentiating between the “response group” and the “non-response group”; Youden’s J statistic was applied. The mean concentrations of serum anti-adalimumab antibodies were 48.89 ± 200.15 ng/mL in the response group and 287.59 ± 444.48 ng/mL in the non-response group (*p* = 0.002). ROC curves were utilized to explore discrimination between the “response group” and the “non-response group” and to determine the cutoff point for anti-adalimumab antibodies, as depicted in Figure 2A. The area under the ROC curve was 0.808 (95% CI: 0.646–0.970) and the optimal combination of sensitivity and specificity was observed with a cutoff level of 7.426 ng/mL, which was identified to predict patient’s response to adalimumab. As depicted in Figure 2B, patients with anti-adalimumab antibody titer over 7.426 ng/mL were less likely to achieve inactive status (*p* = 0.008). Specifically, ninety-three percent of patients with anti-adalimumab antibody titer less than 7.426 ng/mL achieved inactive disease status within 3 years of treatment, whereas only 59% of those with higher antibody titer achieved inactive disease status.

### 2.4. Factors Related to Immunogenicity

To further explore the possible factors which influenced the production of anti-adalimumab antibodies in the present cohort, we looked into the clinical characteristics, laboratory parameters, and treatment regime prior to anti-drug antibody sampling. The level of anti-adalimumab antibodies above and below 7.426 ng/mL was not statistically different between sex, JIA subtypes, or laboratory parameters, as documented in Table 3. Additionally, the *p*-value analyzing the influence of MTX on the production of anti-adalimumab antibodies was not statistically significant (*p* = 0.542), as 48 out of 50 cases received MTX as a concomitant medication in the present cohort.

### 2.5. Role of Anti-Drug Antibodies in Predicting Disease Relapse

Disease relapse is not uncommon among the patients with good initial treatment responses [11]. We compared those who maintained inactive disease status until the last follow-up and those with disease relapse following initial treatment response and found that there was no statistically significant difference in sex, age at diagnosis, duration from JIA diagnosis to adalimumab initiation, JIA subtypes, laboratory parameters, concomitant medications used, or initial joint involvement, as mentioned in Table 4. Levels of anti-adalimumab antibodies were statistically higher among the relapse than the remission groups (79.92 vs. 5.10, *p* = 0.032). In addition, patients within the relapse group were more likely to receive adalimumab treatment beyond an interval of every 2 weeks, as recommended, in comparison to those who remained in remission (*p* = 0.042).

## 3. Discussion

Adalimumab, a potent TNF inhibitor, significantly improves disease control and limited comorbidities in patients with JIA [6,10,13]. While its immunogenicity and the development of anti-drug antibodies is of concern in interfering with its treatment response, our study represents the first study to utilize the presence of anti-adalimumab antibodies as an early marker in predicting therapeutic responses of adalimumab over a 3-year period. We discovered that one-sixth of our JIA patients experienced persisted disease activity under adalimumab treatment. TMJ involvement is a risk for lesser response to adalimumab. In addition, a cutoff value of 7.426 ng/mL for anti-adalimumab antibodies best distinguish between patients who may reach an inactive disease status during a follow-up period of 3 years. On the other hand, patients with higher serum levels of anti-adalimumab antibodies or those receiving adalimumab injections at intervals longer than two weeks during the tapering process are at an increased risk of future disease flare.

Many factors have previously been reported in predicting the treatment responses to adalimumab in JIA patients. Among them, concomitant MTX with adalimumab appeared to decrease disease relapse and reduce treatment failure [10,16]. MTX is recommended in global guidelines as a first-line DMARD to achieve remission or low disease activity and is known to reduce the immunogenicity of adalimumab beyond its known anti-inflammatory effect [8,9,23]. Investigated in a randomized study by *Lovell* et al., it was suggested that the combination of adalimumab and MTX treatment in polyarticular-course juvenile rheumatoid arthritis showed a stronger therapy response at 16 weeks into therapy [10]. For patients with polyarticular JIA in the German BIKER registry, the chance of reaching inactive disease was increased by 85% compared with adalimumab monotherapy, with a risk ratio of 1.85 [24]. However, this finding is not always consistent [10,25]. In the present study, considering that nearly all patients (48 out of 50) in the present cohort received MTX at a stable dose of 2.5 mg per week as a concomitant medication, the influence of MTX on the production of anti-adalimumab antibodies remains unclear.

The immunogenicity of biologics has been a significant concern for the loss of therapeutic response, particularly regarding adalimumab therapy in JIA [16,20]. Specifically, decreased serum level of adalimumab was reported among those with anti-drug antibodies [21,22]. In fact, before adalimumab was approved for JIA treatment, neutralizing responses was already discovered among patients with Crohn’s disease [26]. Despite so, the exact clinical association between anti-adalimumab antibodies and JIA disease manifestation remain inconclusive. For example, while the presence of anti-adalimumab antibodies seemed to relate to the number of relapses [17], as well as treatment failure after one year following adalimumab therapy [18], another study showed that the therapeutic effects were not altered in the presence of anti-drug antibodies [27]. Previous data indicate that anti-adalimumab antibodies are not detectable prior to adalimumab exposure but may be identified as early as 2–3 months after the initiation of treatment [16,22,28]. Considering that the levels of adalimumab were decreased months before clinical loss of function in those who with anti-adalimumab antibodies [20], we utilized anti-adalimumab antibody as a marker to predict treatment response in a 3-year period following treatment. Despite lack of direct measurement of the drug level itself, our data suggest that the presence of anti-adalimumab antibodies at a concentration greater than 7.426 ng/mL following 6 months of treatment is a useful marker capable of predicting its therapeutic response.

The presence of adalimumab has been reported in approximately 18.4% to 45% of JIA patients following treatment, according to previous studies [16,17,20,21]. However, cutoff values for antibody positivity vary significantly between studies. For instance, a cutoff value of 10 AU/mL was considered positive in *Brunelli’s* [22] and *Murias’* studies [29]. Other researchers set their cutoffs at 12 AU/mL in *Leinonen’s* study [30], 0.1 AU/mL in *Skrabl-Baumgartner’s* study [16], and 20 ng/mL in *Burgos-Vargas’s* study [27]. This variability is expected given the differing methodologies across studies, but it does raise questions about the appropriate cutoff for antibody titer in clinical practice. In the present cohort, half of all JIA patients were tested positive for anti-adalimumab antibodies when the cutoff value was set at 0 ng/mL and 34% when we readjusted the cutoff value to be 7.426 ng/mL. Although the presence of antibodies above 0 ng/mL was initially correlated with a higher likelihood of achieving inactive disease status, a cutoff of 7.426 ng/mL was found to be the most effective in distinguishing patients likely to reach inactive disease status over a 3-year period. To the best of our knowledge, this is the first study to investigate and propose a cutoff value of anti-adalimumab antibodies for therapeutic outcome prediction.

ERA is the leading JIA subtype in Taiwan [14,31]. Different subtypes of JIA were known to have variable outcomes with ERA appearing to have a relatively poor response to therapies or a low percentage of clinical remission [15,32]. The therapeutic effect of adalimumab in ERA was successfully demonstrated in 2015 [27]; however, direct comparison of its efficacy in treating ERA patients and patients with other JIA subtypes is limited. Data from *Shih* et al. suggested a tendency of persistent active disease in patient with ERA, despite anti-TNF biologics treatment [14]. A recent report from *Shipa* et al. showed that 29 out of 80 (36.25%) ERA patients discontinued adalimumab due to treatment failure or adverse drug reactions and a higher rate of discontinuation with adalimumab was found for those with higher baseline CRP level in patients with ERA [33]. Taking advantage of the relatively high prevalence of ERA in our cohort, we compared the response of ERA and non-ERA to adalimumab treatment and discovered that the chance of achieving inactive disease by adalimumab in JIA patients with ERA and other JIA subtypes were fairly comparable. This suggested that despite a shorter experience of adalimumab in ERA treatment, a clinician may expect a non-inferior treatment response as compared to that of polyarticular course JIA.

Early therapeutic responses to adalimumab [11], lack of uveitis [34], and sacroiliac joint involvement [14] were also reported with a more favorable response to adalimumab in JIA. In our study, we found that non-responders were more likely to have TMJ involvement initially. This finding is compatible with previous studies, which showed that TMJ arthritis was associated with a severe disease course and a decreased probability of achieving remission [35].

Disease relapse following an initial response is a stressful and unpleasant event for both the patients and clinicians. *Doeleman* et al. have reported that lower adalimumab concentration may increase the chance of disease relapse in JIA patients receiving adalimumab treatment [18]. In addition, the presence of anti-adalimumab antibodies has also been shown to correlate with the number of relapses [17]. In the present study, we discovered that while higher serum levels of anti-adalimumab antibodies were noted between the remission and the relapsed groups, prolonging the injection interval beyond 2 weeks between adalimumab injections, as a common tapering strategy in real world practice [36,37], was also essential in determining future disease. That is to say, regardless of the presence of anti-adalimumab antibodies, disease relapse should be closely monitored during the tapering process.

There are several limitations in our study. Lack of direct measurement of adalimumab level in patient serum is perhaps the major limitation in our study. However, although we were unable to distinguish neutralizing antibodies from non-neutralizing antibodies due to the nature of the methodology we applied for antibody measurement, utilizing the same measurement technique, *Judit* have successfully shown that the presence of anti-drug antibodies with the same methodology correlated negatively with the level of adalimumab [38]. Despite the fact that the exact drug levels were unknown, the presence of anti-adalimumab antibodies itself after 6 months remained a valuable marker for treatment outcome prediction. Next, our result is limited by the lack of long-term therapeutic effect as the follow-up period was set at 3 years. In addition, a small sample size is an unavoidable limitation in pediatric rheumatic disease research conducted in a single-center setting. Due to the lack of comprehensive records, including the juvenile arthritis disease activity score, visual analogue scale, and parent global assessment—stemming from variations in individual physicians’ practices—these parameters were not included in the risk factor evaluation. Finally, although physicians reached a consensus to carefully extend the administration interval of biologics in patients with inactive disease in our institute, there was no pre-specified protocol. The timing of when to start tapering or the schedule of biologics tapering may also bias the analysis for disease relapse.

## 4. Materials and Methods

### 4.1. Study Subjects

Patients with JIA who received adalimumab treatment with regular follow ups over a 3-year period between 2011 to 2022, in a tertiary medical center in Taiwan, were enrolled. The inclusion criteria for the enrolled patients required that they meet the International League of Associations for Rheumatology (ILAR) classification criteria for JIA and be diagnosed before 16 years of age [1]. Patients with a history of other joint or bone disorders (e.g., primary osteoarthritis, bone cancer), metabolic syndromes such as diabetes, overlapping rheumatic disorders like systemic lupus erythematosus, Sjögren’s disease, juvenile dermatomyositis, or pregnancy were excluded. Clinical data including the information of sex, age, disease duration, JIA subtypes, involved joints, and medication history, as well as laboratory data, were carefully reviewed and collected.

### 4.2. Ethics

This study is in compliance with the Declaration of Helsinki and the study design was approved by the institutional review board of Chang Gung Medical Foundation (IRB No.: 202202170A3). Informed consent forms were obtained from all participating patients and/or their guardians.

### 4.3. Treatment Response

Disease status was evaluated according to the Wallace criteria at 0, 6, 12, 18, 24, 30, and 36 months after initiation of adalimumab treatment [39]. Patients were categorized into a “response group” if they achieved inactive disease status any time during the 3 years of follow-up since the initiation of adalimumab. Within the response group, patients who maintained inactive status until the last follow-up were classified as “remission”. Patients who did not fulfill the definition of remission following an initial inactive disease status were categorized as “relapse”.

### 4.4. Anti-Adalimumab Antibodies

Serum samples were collected 6 months after initiation of adalimumab therapy. Anti-adalimumab antibodies were measured utilizing a validated commercial enzyme-linked immunosorbent assay (Shikari^®^ (S-ATA)) purchased from Matriks biotek (Ankara, Turkey) [38,40,41]. The samples were processed and tested according to the manufacturer’s instructions. A level of anti-adalimumab antibodies more than 0 ng/mL is considered detectable.

### 4.5. Statistical Analysis

Baseline characteristics were analyzed using descriptive statistics. Binary variables were assessed using chi-square test and Fisher’s exact test. Continuous variables were analyzed using a Mann–Whitney U Test. The areas under curves (AUCs) of the receiver operating characteristic (ROC) curves were to explore discriminations between the response and non-response groups and determine the cutoff value for anti-adalimumab antibodies. The statistical significance was set at *p* value < 0.05. All statistical analyses were conducted using SPSS version 25.

## 5. Conclusions

In conclusion, our result suggested that anti-adalimumab antibody may be used as a predictor of therapeutic response in patients treated with adalimumab up to three years. Additionally, we identified an optimal cutoff value for anti-adalimumab antibodies to differentiate patients likely to achieve an inactive disease status within three years.

## Figures and Tables

**Table 1:** Demographic information and baseline characteristics of the patients.

	*n* = 65
Male subjects, *n* (%)	40 (61.5)
Age at diagnosis, mean ± SD, years	10.47 ± 3.90
Disease duration, mean ± SD, years	7.97 ± 5.53
Duration between diagnosis and adalimumab initiation, mean ± SD, years	3.28 ± 4.97
Duration of adalimumab use, mean ± SD, years	2.64 ± 0.56
JIA subtypes, *n* (%)	
Oligoarthritis	1 (1.5)
Seronegative polyarthritis	16 (24.6)
Seropositive polyarthritis	5 (7.7)
Enthesitis related arthritis	42 (64.6)
Undifferentiated arthritis	1 (1.5)
Laboratory parameters, *n* (%)	
ANA positive (≥1:80) *	4 (6.6)
RF †	5 (7.9)
HLA-B27 ‡	40 (65.6)
Previous Medications, *n* (%)	
NSAIDs	62 (95.4)
Systemic steroids	55 (84.6)
Intra-articular glucocorticoid	25 (38.5)
Methotrexate	56 (86.2)
Sulfasalazine	30 (46.2)
Other TNF-α (Etanercept)	4 (6.2)

* ANA was tested in 60 of our patients. † RF was tested in 63 of our patients. ‡ HLA-B27 was tested in 61 of our patients.

**Table 2:** Difference between JIA patients with responders and non-responders within the 3-year period following adalimumab treatment.

	Response Group *n* = 54	Non-Response Group *n* = 11	*p*-Value
Male subjects, *n* (%)	34 (63.0)	6 (54.5)	0.601
Age at diagnosis, mean ± SD, years	10.53 ± 3.98	10.08 ± 3.62	0.791
Duration between diagnosis and adalimumab initiation, mean ± SD, years	2.71 ± 3.65	6.52 ± 9.27	0.183
Duration of adalimumab use, mean ± SD, years	2.61 ± 5.82	2.76 ± 0.46	0.972
JIA subtypes, *n* (%)			0.187
Oligoarthritis	1 (1.9)	0	
Seronegative polyarthritis	13 (24.1)	3 (27.3)	
Seropositive polyarthritis	5 (9.3)	0	
ERA	35 (64.8)	7 (63.6)	
Undifferentiated arthritis	0	1 (9.1)	
ERA/non-ERA	35/19	7/4	1.000
Laboratory parameters, *n* (%)			
ANA positive (≥1:80) $	4 (8.1)	0	1.000
RF †	4 (7.7)	1 (9.1)	1.000
HLA-B27 ‡	32 (64.0)	8 (72.7)	0.581
CRP, mean ± SD, mg/L	35.68 ± 24.35	35.50 ± 27.27	0.340
ESR, mean ± SD, mm/h	29.37 ± 17.86	31.45 ± 25.15	0.971
Concomitant medications used, *n* (%)			
NSAIDs	13 (24.1)	3 (27.3)	1.000
Systemic steroids	5 (9.3)	3 (27.3)	0.126
Methotrexate	51 (94.4)	11 (100)	1.000
Sulfasalazine	8 (14.8)	4 (36.4)	0.194
Initial joint involvement, *n*			
TMJs	5	4	0.038 *
SI joints	8	3	0.379
Enthesitis, *n* (%)	7	0	0.592
Uveitis, *n* (%)	4	0	1.000
Anti-adalimumab antibodies			
Detectable, *n* (%)	17 (31.5)	8 (73.7)	0.023 *
Concentrations, mean ± SD, ng/mL	48.89 ± 200.15	287.59 ± 444.48	0.002 *

* *p*-value < 0.05 is considered statistically significant. $ ANA was tested in 49 of the responders and 11 of the non-responders. † RF was tested in 52 of the responders and 11 of the non-responders. ‡ HLA-B27 was tested in 50 of the responders and 11 of the non-responders.

**Table 3:** Difference between JIA patients with anti-adalimumab antibodies above and below 7.426 ng/mL following 6 months of treatment.

	Antibodies ≥ 7.426 ng/mL *n* = 17	Antibodies < 7.426 ng/mL *n* = 33	*p*-Value
Male subjects, *n* (%)	8 (47.1)	23 (69.7)	0.118
Age at diagnosis, mean ± SD, years	10.50 ± 3.14	10.36 ± 4.14	0.900
Duration between diagnosis and adalimumab initiation, mean ± SD, years	4.43 ± 7.54	2.63 ± 3.46	0.493
Duration of adalimumab use, mean ± SD, years	2.61 ± 0.53	2.59 ± 0.64	0.935
JIA subtypes, *n* (%)			0.111
Oligoarthritis	0	1 (3.0)	
Seronegative polyarthritis	6 (35.3)	9 (27.3)	
Seropositive polyarthritis	2 (11.8)	0	
ERA	8 (47.1)	23 (69.7)	
Undifferentiated arthritis	1 (5.9)	0	
ERA/non-ERA	8/9	23/10	0.118
Laboratory parameters, *n* (%)			
ANA positive $ (≥1:80)	2 (12.5)	2 (6.7)	0.602
RF †	2 (12.5)	0	0.106
HLA-B27 ‡	8 (50.0)	22 (73.3)	0.114
CRP, mean ± SD, mg/L	36.45 ± 31.01	35.14 ± 20.24	0.095
ESR, mean ± SD, mm/h	24.00 ± 17.31	30.71 ± 17.89	0.129
Concomitant medications between 0–6 month, *n* (%)			
NSAIDs	15 (88.2)	32 (97.0)	0.264
Oral Steroids	14 (82.4)	26 (78.8)	1.000
Methotrexate	17 (100)	31 (93.9)	0.542
Sulfasalazine	7 (41.2)	15 (45.5)	0.703
Initial joint involvement, *n*			
TMJs	3	3	0.396
SI joints	2	7	0.699
Enthesitis, *n* (%)	0	7 (21.2)	0.080
Uveitis, *n* (%)	1 (5.9)	3 (9.1)	1.000

$ ANA was tested in 16 of the patients with antibodies level above 7.426 ng/mL and 30 of the patients with antibodies level below 7.426 ng/mL. † RF was tested in 16 of the patients with antibodies level above 7.426 ng/mL and 32 of the patients with antibodies level below 7.426 ng/mL. ‡ HLA-B27 was tested in 16 of the patients with antibodies level above 7.426 ng/mL and 30 of the patients with antibodies level below 7.426 ng/mL.

**Table 4:** Difference between the JIA patients with disease remission or relapse after achieving inactive disease status.

	Remission *n* = 22	Relapse *n* = 32	*p*-Value
Male subjects, *n* (%)	16 (72.7)	18 (56.3)	0.218
Age at diagnosis, mean ± SD, years	10.36 ± 3.79	10.66 ± 4.17	0.753
Duration between diagnosis and adalimumab initiation, mean ± SD, years	2.74 ± 3.58	2.70 ± 3.77	0.677
Duration of adalimumab use, mean ± SD, years	2.50 ± 0.55	2.79 ± 0.44	0.000 *
JIA subtypes, *n* (%)			0.854
Oligoarthritis	0	1 (3.1)	
Seronegative polyarthritis	5 (22.7)	8 (25.0)	
Seropositive polyarthritis	2 (9.1)	3 (9.4)	
ERA	15 (68.2)	20 (62.5)	
Undifferentiated arthritis	0	0	
ERA/non-ERA	15/7	20/12	0.667
Laboratory parameters, *n* (%)			
ANA positive (≥1:80) $	0 (0.0)	4 (14.3)	0.125
RF †	2 (9.1)	2 (6.7)	1.000
HLA-B27 ‡	14 (66.7)	18 (62.1)	0.774
CRP, mean ± SD, mg/L	30.42 ± 18.78	39.68 ± 27.55	0.224
ESR, mean ± SD, mm/h	26.48 ± 13.98	31.40 ± 20.11	0.559
Concomitant medications used, *n* (%)			
NSAIDs	2 (9.1)	11 (34.4)	0.051
Systemic steroids	0	5 (15.6)	0.072
Methotrexate	19 (86.4)	32 (100)	0.062
Sulfasalazine	2 (9.1)	6 (18.8)	0.449
Initial joint involvement, *n*			
TMJs	4	1	0.146
SI joints	2	6	0.449
Enthesitis, *n* (%)	2 (9.1)	5 (15.6)	0.687
Uveitis, *n* (%)	2 (9.1)	2 (6.3)	1.000
Anti-adalimumab antibodies			
Antibodies ≥ 7.426 ng/mL, *n* (%)	2/17 (11.8)	8/24 (33.3)	0.152
Concentrations, mean ± SD, ng/mL	5.10 ± 16.72	79.92 ± 258.95	0.032 *
Adalimumab schedule, *n*			
Every other week	13	10	0.042 *
Prolonged interval (>2 weeks)	9	22	

* *p*-value < 0.05 is considered statistically significant. $ ANA was tested in 21 of the remission group and 28 of the relapse group. † RF was tested in 22 of the remission group and 30 of the relapse group. ‡ HLA-B27 was tested in 21 of the remission group and 29 of the relapse group.

## Data Availability

The data presented in this study are available from the corresponding author upon request.

## Abbreviations

ANA	Antinuclear antibody
AUC	Area under curve
CRP	C-reactive protein
DMARDs	Disease-modifying anti-rheumatic drugs
ERA	Enthesitis-related arthritis
ESR	Erythrocyte sedimentation rate
HLA-B27	Human leukocyte antigen B27
JIA	Juvenile idiopathic arthritis
MTX	Methotrexate
NSAIDs	Non-steroidal anti-inflammatory drugs
RF	Rheumatoid factor
SI	Sacroiliac
TMJ	Temporomandibular joint
TNF-α	Tumor necrosis factor alpha

## References

[1] Petty R.E., Southwood T.R., Manners P., Baum J., Glass D.N., Goldenberg J., He X., Maldonado-Cocco J., Orozco-Alcala J., Prieur A.M. (2004). International League of Associations for Rheumatology classification of juvenile idiopathic arthritis: Second revision, Edmonton, 2001. J. Rheumatol..

[2] Prakken B., Albani S., Martini A. (2011). Juvenile idiopathic arthritis. Lancet.

[3] Haverman L., Verhoof E.J., Maurice-Stam H., Heymans H.S., Gerlag D.M., van Rossum M.A., Grootenhuis M.A. (2012). Health-related quality of life and psychosocial developmental trajectory in young female beneficiaries with JIA. Rheumatology.

[4] Thorne J.E., Woreta F., Kedhar S.R., Dunn J.P., Jabs D.A. (2007). Juvenile idiopathic arthritis-associated uveitis: Incidence of ocular complications and visual acuity loss. Am. J. Ophthalmol..

[5] Giancane G., Muratore V., Marzetti V., Quilis N., Benavente B.S., Bagnasco F., Alongi A., Civino A., Quartulli L., Consolaro A. (2019). Disease activity and damage in juvenile idiopathic arthritis: Methotrexate era versus biologic era. Arthritis Res. Ther..

[6] Ramanan A.V., Dick A.D., Jones A.P., McKay A., Williamson P.R., Compeyrot-Lacassagne S., Hardwick B., Hickey H., Hughes D., Woo P. (2017). Adalimumab plus Methotrexate for Uveitis in Juvenile Idiopathic Arthritis. N. Engl. J. Med..

[7] Minden K., Niewerth M., Zink A., Seipelt E., Foeldvari I., Girschick H., Ganser G., Horneff G. (2012). Long-term outcome of patients with JIA treated with etanercept, results of the biologic register JuMBO. Rheumatology.

[8] Ringold S., Angeles-Han S.T., Beukelman T., Lovell D., Cuello C.A., Becker M.L., Colbert R.A., Feldman B.M., Ferguson P.J., Gewanter H. (2019). 2019 American College of Rheumatology/Arthritis Foundation Guideline for the Treatment of Juvenile Idiopathic Arthritis: Therapeutic Approaches for Non-Systemic Polyarthritis, Sacroiliitis, and Enthesitis. Arthritis Rheumatol..

[9] Onel K.B., Horton D.B., Lovell D.J., Shenoi S., Cuello C.A., Angeles-Han S.T., Becker M.L., Cron R.Q., Feldman B.M., Ferguson P.J. (2022). 2021 American College of Rheumatology Guideline for the Treatment of Juvenile Idiopathic Arthritis: Therapeutic Approaches for Oligoarthritis, Temporomandibular Joint Arthritis, and Systemic Juvenile Idiopathic Arthritis. Arthritis Care Res..

[10] Lovell D.J., Ruperto N., Goodman S., Reiff A., Jung L., Jarosova K., Nemcova D., Mouy R., Sandborg C., Bohnsack J. (2008). Adalimumab with or without methotrexate in juvenile rheumatoid arthritis. N. Engl. J. Med..

[11] Lovell D.J., Brunner H.I., Reiff A.O., Jung L., Jarosova K., Nemcova D., Mouy R., Sandborg C., Bohnsack J.F., Elewaut D. (2020). Long-term outcomes in patients with polyarticular juvenile idiopathic arthritis receiving adalimumab with or without methotrexate. RMD Open.

[12] Horneff G., Klein A., Klotsche J., Minden K., Huppertz H.I., Weller-Heinemann F., Kuemmerle-Deschner J., Haas J.P., Hospach A. (2016). Comparison of treatment response, remission rate and drug adherence in polyarticular juvenile idiopathic arthritis patients treated with etanercept, adalimumab or tocilizumab. Arthritis Res. Ther..

[13] Takei S., Iwata N., Kobayashi I., Igarashi T., Yoshinaga Y., Matsubara N., Sunaga N., Ito A., Yokota S. (2021). Safety and effectiveness of adalimumab in Japanese patients with juvenile idiopathic arthritis: Results from a real-world postmarketing study. Mod. Rheumatol..

[14] Shih Y.J., Yang Y.H., Lin C.Y., Chang C.L., Chiang B.L. (2019). Enthesitis-related arthritis is the most common category of juvenile idiopathic arthritis in Taiwan and presents persistent active disease. Pediatr. Rheumatol. Online J..

[15] Glerup M., Rypdal V., Arnstad E.D., Ekelund M., Peltoniemi S., Aalto K., Rygg M., Toftedal P., Nielsen S., Fasth A. (2020). Long-Term Outcomes in Juvenile Idiopathic Arthritis: Eighteen Years of Follow-Up in the Population-Based Nordic Juvenile Idiopathic Arthritis Cohort. Arthritis Care Res..

[16] Skrabl-Baumgartner A., Erwa W., Muntean W., Jahnel J. (2015). Anti-adalimumab antibodies in juvenile idiopathic arthritis: Frequent association with loss of response. Scand. J. Rheumatol..

[17] Marino A., Real-Fernandez F., Rovero P., Giani T., Pagnini I., Cimaz R., Simonini G. (2018). Anti-adalimumab antibodies in a cohort of patients with juvenile idiopathic arthritis: Incidence and clinical correlations. Clin. Rheumatol..

[18] Doeleman M.J.H., de Roock S., El Amrani M., van Maarseveen E.M., Wulffraat N.M., Swart J.F. (2021). Association of adalimumab trough concentrations and treatment response in patients with juvenile idiopathic arthritis. Rheumatology.

[19] Trotta M.C., Alfano R., Cuomo G., Romano C., Gravina A.G., Romano M., Galdiero M., Montemurro M.V., Giordano A., D’Amico M. (2022). Comparison of Timing to Develop Anti-Drug Antibodies to Infliximab and Adalimumab Between Adult and Pediatric Age Groups, Males and Females. J. Pediatr. Pharmacol. Ther..

[20] Skrabl-Baumgartner A., Seidel G., Langner-Wegscheider B., Schlagenhauf A., Jahnel J. (2019). Drug monitoring in long-term treatment with adalimumab for juvenile idiopathic arthritis-associated uveitis. Arch. Dis. Child..

[21] Nassar-Sheikh Rashid A., Schonenberg-Meinema D., Bergkamp S.C., Bakhlakh S., de Vries A., Rispens T., Kuijpers T.W., Wolbink G., van den Berg J.M. (2021). Therapeutic drug monitoring of anti-TNF drugs: An overview of applicability in daily clinical practice in the era of treatment with biologics in juvenile idiopathic arthritis (JIA). Pediatr. Rheumatol. Online J..

[22] Brunelli J.B., Silva C.A., Pasoto S.G., Saa C.G.S., Kozu K.T., Goldenstein-Schainberg C., Leon E.P., Vendramini M.B.G., Fontoura N., Bonfa E. (2020). Anti-adalimumab antibodies kinetics: An early guide for juvenile idiopathic arthritis (JIA) switching. Clin. Rheumatol..

[23] Doeleman M.J.H., van Maarseveen E.M., Swart J.F. (2019). Immunogenicity of biologic agents in juvenile idiopathic arthritis: A systematic review and meta-analysis. Rheumatology.

[24] Thiele F., Klein A., Klotsche J., Windschall D., Dressler F., Kuemmerle-Deschner J., Minden K., Foeldvari I., Foell D., Mrusek S. (2023). Biologics with or without methotrexate in treatment of polyarticular juvenile idiopathic arthritis: Effectiveness, safety and drug survival. Rheumatology.

[25] Klein A., Becker I., Minden K., Foeldvari I., Haas J.P., Horneff G. (2019). Adalimumab versus adalimumab and methotrexate for the treatment of juvenile idiopathic arthritis: Long-term data from the German BIKER registry. Scand. J. Rheumatol..

[26] West R.L., Zelinkova Z., Wolbink G.J., Kuipers E.J., Stokkers P.C., van der Woude C.J. (2008). Immunogenicity negatively influences the outcome of adalimumab treatment in Crohn’s disease. Aliment. Pharmacol. Ther..

[27] Burgos-Vargas R., Tse S.M., Horneff G., Pangan A.L., Kalabic J., Goss S., Unnebrink K., Anderson J.K. (2015). A Randomized, Double-Blind, Placebo-Controlled Multicenter Study of Adalimumab in Pediatric Patients with Enthesitis-Related Arthritis. Arthritis Care Res..

[28] Imagawa T., Takei S., Umebayashi H., Yamaguchi K., Itoh Y., Kawai T., Iwata N., Murata T., Okafuji I., Miyoshi M. (2012). Efficacy, pharmacokinetics, and safety of adalimumab in pediatric patients with juvenile idiopathic arthritis in Japan. Clin. Rheumatol..

[29] Murias S., Alcobendas R., Pascual-Salcedo D., Remesal A., Peralta J., Merino R. (2014). Anti-adalimumab antibodies in paediatric rheumatology patients: A pilot experience. Rheumatology.

[30] Leinonen S.T., Aalto K., Kotaniemi K.M., Kivela T.T. (2017). Anti-adalimumab antibodies in juvenile idiopathic arthritis-related uveitis. Clin. Exp. Rheumatol..

[31] Shen C.C., Yeh K.W., Ou L.S., Yao T.C., Chen L.C., Huang J.L. (2013). Clinical features of children with juvenile idiopathic arthritis using the ILAR classification criteria: A community-based cohort study in Taiwan. J. Microbiol. Immunol. Infect..

[32] Weiss P.F., Beukelman T., Schanberg L.E., Kimura Y., Colbert R.A. (2012). Enthesitis-related arthritis is associated with higher pain intensity and poorer health status in comparison with other categories of juvenile idiopathic arthritis: The Childhood Arthritis and Rheumatology Research Alliance Registry. J. Rheumatol..

[33] Shipa M.R., Heyer N., Mansoor R., Deakin C.T., Madenidou A.V., Bouraioui A., Fisher C., Leandro M., Ciurtin C., Sen D. (2022). Adalimumab or etanercept as first line biologic therapy in enthesitis related arthritis (ERA)—A drug-survival single centre study spanning 10 years. Semin. Arthritis Rheum..

[34] Kostik M.M., Gaidar E.V., Sorokina L.S., Avrusin I.S., Nikitina T.N., Isupova E.A., Chikova I.A., Korin Y.Y., Orlova E.D., Snegireva L.S. (2022). Uveitis Is a Risk Factor for Juvenile Idiopathic Arthritis’ Significant Flare in Patients Treated with Biologics. Front. Pediatr..

[35] Artamonov A.K., Kaneva M.A., Gordeeva N.A., Sorokina L.S., Kostik M.M. (2023). Temporomandibular Joint Involvement in Juvenile Idiopathic Arthritis: The Results from a Retrospective Cohort Tertial Center Study. Life.

[36] Liao C.H., Chiang B.L., Yang Y.H. (2021). Tapering of Biological Agents in Juvenile ERA Patients in Daily Clinical Practice. Front. Med..

[37] Horton D.B., Onel K.B., Beukelman T., Ringold S. (2017). Attitudes and Approaches for Withdrawing Drugs for Children with Clinically Inactive Nonsystemic JIA: A Survey of the Childhood Arthritis and Rheumatology Research Alliance. J. Rheumatol..

[38] Judit Szántó K., Madácsy T., Kata D., Ferenci T., Rutka M., Bálint A., Bor R., Fábián A., Milassin Á., Jójárt B. (2021). Advances in the optimization of therapeutic drug monitoring using serum, tissue and faecal anti-tumour necrosis factor concentration in patients with inflammatory bowel disease treated with TNF-α antagonists. Expert Opin. Biol. Ther..

[39] Wallace C.A., Ruperto N., Giannini E., Childhood A., Rheumatology Research A., Pediatric Rheumatology International Trials O., Pediatric Rheumatology Collaborative Study G. (2004). Preliminary criteria for clinical remission for select categories of juvenile idiopathic arthritis. J. Rheumatol..

[40] Sam M.J., Connor S.J., Ng W.W., Toong C.M. (2020). Comparative Evaluation of 4 Commercially Available ELISA Kits for Measuring Adalimumab and Anti-adalimumab Antibodies. Ther. Drug Monit..

[41] Kui R., Gál B., Gaál M., Kiss M., Kemény L., Gyulai R. (2016). Presence of antidrug antibodies correlates inversely with the plasma tumor necrosis factor (TNF)-α level and the efficacy of TNF-inhibitor therapy in psoriasis. J. Dermatol..

